# Green Biosynthesis of Selenium Nanoparticles Using Orange Peel Waste: Characterization, Antibacterial and Antibiofilm Activities against Multidrug-Resistant Bacteria

**DOI:** 10.3390/life12060893

**Published:** 2022-06-15

**Authors:** Salem S. Salem, Mona Shaban E. M. Badawy, Abdulaziz A. Al-Askar, Amr Abker Arishi, Fathy M. Elkady, Amr H. Hashem

**Affiliations:** 1 Botany and Microbiology Department, Faculty of Science, Al-Azhar University, Nasr City, Cairo 11884, Egypt; 2 Department of Microbiology and Immunology, Faculty of Pharmacy (Girls), Al-Azhar University, Nasr City, Cairo 11884, Egypt; mony.badawe@yahoo.com; 3 Department of Botany and Microbiology, Faculty of Science, King Saud University, Riyadh 12372, Saudi Arabia; 4 School of Molecular Sciences, The University of Western Australia, Perth, WA 6009, Australia; 22650755@student.uwa.edu.au; 5 Microbiology and Immunology Department, Faculty of Pharmacy (Boys), Al-Azhar University, Nasr City, Cairo 11884, Egypt; fathyelkady2426.el@azhar.edu.eg

**Keywords:** orange peel waste, biosynthesis, selenium nanoparticles, antibacterial activity, antibiofilm activity

## Abstract

There is an increase of pathogenic multidrug-resistant bacteria globally due to the misuse of antibiotics. Recently, more scientists used metal nanoparticles to counteract antibacterial resistance. In this study, orange peel waste (OPW) was used for selenium nanoparticles’ (Se-NPs) biosynthesis through the green and ecofriendly method, and their applications as antibacterial and antibiofilm agents. Green biosynthesized Se-NPs were characterized using FTIR, XRD, SEM, EDAX, and TEM. Characterization results revealed that biosynthesized Se-NPs were highly crystalline, spherical, and polydisperse, and had sizes in the range of 16–95 nm. The biosynthesized Se-NPs were evaluated as antibacterial and antibiofilm activities against multidrug-resistant bacteria. Results illustrated that Se-NPs exhibited potential antibacterial activity against Gram-positive bacteria (*S. aureus* ATCC 29213 and biofilm-producing clinical isolates of *S. aureus*) and Gram-negative bacteria (*Pseudomonas aeruginosa* PAO1, MDR, biofilm, and quorum-sensing and producing clinical isolates of MDR *P. aeruginosa,* MDR *E. coli*, and *K. pneumonia*). Moreover, results illustrated that *S. aureus* ATCC 29213 was the most sensitive bacteria to Se-NPs at 1000 µg/mL, where the inhibition zone was 35 mm and MIC was 25 µg/mL. Furthermore, Se-NPs at 0.25 and 0.5 MIC decreased the biofilm significantly. The largest inhibition of biofilm was noticed in MDR *K. pneumonia*, which was 62% and 92% at 0.25 and 0.5 MIC, respectively. In conclusion, Se-NPs were successfully biosynthesized using OPW through the green method and had promising antibacterial and antibiofilm activity against multidrug-resistant bacteria, which can be used later in fighting resistant bacteria.

## 1. Introduction

Antimicrobial resistance (AMR) to antibiotics allows bacteria to evolve to diverse methods to shield themselves from the antibiotics’ effects. Although not a new problem but rather a long-standing property of bacteria, the AMR dilemma has been exacerbated by widespread overuse and abuse of existing antibiotics in healthcare, agriculture, and cattle, among other contexts [1]. Unfortunately, standard antibiotic-based therapies are unable to keep up with bacterial development, resulting in an unclear future. AMR will cause more than 10 million deaths worldwide by 2050, at a cost of more than USD 100 trillion [2]. Furthermore, microbial biofilms are important in many diseases and biofilm-related features may impart significant levels of antibiotic resistance in microbial populations [3]. Antibacterial drugs and immune response effectors may be hampered by the biofilm matrix, which can operate as a mechanical barrier. Bacteria can also become extremely resistant to antibiotics as a result of food deficiency or the establishment of a permanent but non-growing phenotype that permits microbial cells to deal well with environmental challenges, such as antibiotic exposure [4]. This situation necessitates immediate attention and a more creative approach to the development of novel, effective, and safe antimicrobial drugs using nanotechnology.

Nanotechnology is assumed to be the subsequent industrial revolution and is considered to have a tremendous effect on the community, economics, and the common world [5,6,7]. This field possesses great effectiveness in various sites including infection control, biomedicine, industry, wastewater treatment, and agriculture [8,9,10,11,12,13,14,15]. The environmentally friendly approach for the biosynthesis of the nanoparticle is an opportunity for appling it safely in medical fields [16,17,18]. Recently, green biosynthesis, eco-friendly, safe, clean, and cost-effective are recommended for nanometals’ preparation today [19,20,21]. Antimicrobial nanoparticles have numerous potential benefits over traditional antibiotics, including several mechanisms of action on bacteria, easy and low-cost production, and great stability [22]. In vitro and in vivo studies have shown that antibacterial nanoparticles can suppress infection [23,24]. Furthermore, antimicrobial nanoparticles may harm bacteria in a variety of ways, making it harder for bacteria to acquire resistance [25].

The mechanism of action of metal nanoparticles as antimicrobial agents is attributed to different mechanisms: (1) metabolic interference via intercellular adenosine triphosphate (ATP) concentrations, (2) intracellular reactive oxygen species (ROS) concentration modulation, (3) bacterial membrane depolarization, and (4) bacterial membrane interruption. ATP is an internal energy that is used by all living organisms. It is an important source of energy for respiration and metabolism [26]. Virulence factors of bacteria, such as sluggish drug absorption and rapid efflux, biofilm development, and intracellular bacterial parasitism, have been proven to be overcome by these nanoparticles [27]. In the current study, orange peel waste was used for the biosynthesis of Se-NPs through a green and eco-friendly method. Additionally, we explored the antimicrobial and antibiofilm activities of green biosynthesized Se-NPs using OPW for the first time.

## 2. Materials and Methods

### 2.1. Preparation of Orange Peel Waste (OPW) Extract

OPW was collected from local markets in Giza, Egypt. The collected samples were transported to the laboratory and processed immediately. OPW without disease symptoms was selected and then washed twice with distilled water (D.W). Then, the peel was cut into small pieces (~1 cm); 200 g was placed in 1000 mL of deionized water in a 2 L conical flask and mixed in a blender (mixer) at 1000 rpm for 3 min, and then filtered using Whatman no. 1 filter paper; and then collected into a sterilized bottle; and kept at 4 °C until further use [28,29].

### 2.2. Biosynthesis of Se-NPs

OPW was used for the green biosynthesis Se-NPs through the ecofriendly method. OPW extract (10 mL) was added to 90 mL of 2 mM Na_2_SeO_3_, where a combination was prepared. For the control sample, 10 mL of D.W was added to 90 mL of 2 mM Na_2_SeO_3_. Both flasks were incubated in the rotary shaker for 3 h in the dark to obtain a homogenous mixture. The generated Se-NPs were then separated and purified using D.W and centrifugation. Dried Se-NPs were stored at room temperature for further analyses.

### 2.3. Characterization of Se-NPs

Characterization of Se-NPs was carried out using different instrumental analytical methods. During the incubation phase, changes in solution color were used to visually examine the development of Se-NPs. The production of Se-NPs by the OPW extract was studied using UV–Vis spectra (JENWAY-6305 Spectrophotometer) at frequencies of 200–800 nm. Furthermore, using a Spectrum Two-IR Spectrometer (Perkin-Elmer Inc., Shelton, CT, USA), the distinctive functional groups contained in produced Se-NPs molecules were investigated. Potassium bromide was added to samples before being put onto high-pressure discs. To produce FTIR spectra, these discs were detected in the 400–4000 cm^−1^ range. To achieve a suitable signal quality, all spectra were collected at a 4 cm^−1^ resolution by collecting 32 scans. Additionally, the crystalline structure of Se-NPs was determined using XRD analysis. A Diano X-ray diffractometer (Philips) with a CuK radiation source (λ = 0.15418 nm) activated at 45-kV, as well as a generator-PW1930 and a goniometer-PW1820, were used to study the XRD pattern of the produced Se-NPs. Furthermore, TEM was used for detecting the shape and size of the biosynthesized Se-NPs. The Ultra-High Resolution TEM (JEOL-2010, Japan) with a voltage of 200 kV was employed. A drop of the particle solution was placed on a carbon-coated copper grid and dried under a light to prepare TEM grids. Moreover, SEM analysis (SEM, ZEiSS, EVO-MA 10, Oberkochen, Germany) was used to detect the surface morphology and size of the synthesized Se-NPs. The elemental composition, purity, and dispersal of the nanoparticle-forming elements were investigated using EDX-BRUKER, Nano-GmbH, (D-12489, M-410) Germany.

### 2.4. Antibacterial Activity

#### 2.4.1. Agar Well Diffusion Method

The antibacterial activity of biosynthesized Se-NPs was evaluated a0gainst Gram-negative bacteria (*Pseudomonas aeruginosa* PAO1, MDR, biofilm, and quorum-sensing and producing clinical isolates of MDR *P. aeruginosa,* MDR *E. coli,* and *K. pneumonia*) and Gram-positive bacteria (*S. aureus* ATCC 29213, MDR clinical isolates, and biofilm-producing clinical isolates of *S. aureus*) using the agar well diffusion method [5]. Briefly, 100 µL/mL of Se-NPs (1000 µg/mL) was put into a well (6 mm) prepared previously on Muller–Hinton agar media streaked with purified bacterial species (0.5 McFarland). Additionally, the orange peel has been used alone as a control sample to investigate its antibacterial activity. The inoculated plates were incubated at 37 ± 2 °C for 24 h. The inhibition zone (mm) for each well was measured separately.

#### 2.4.2. Determination of Minimum Inhibitory Concentrations (MIC) of Se-NPs

The broth microdilution method was used to determine the MIC of Se-NPs. Double-fold serial dilution of Se-NPs was carried out. A volume of 5 μL of pure bacterial species equivalent to a 0.5 McFarland standard was injected into Se-NPs and incubated at 37 °C for 24 h. [30]. Then, 5 μL of indicator resazurin (made by dissolving 0.016 g in 100 mL of sterile DW) was added to each of the 96 wells and incubated in the dark. After the incubation period, the color change was examined, where a change of the color from purple to pink or colorlessness indicates a direct indication of bacterial metabolic activity. The MIC value was detectedas the lowest concentration at which the color change occurred. The MIC of the test material and the bacterial strain were calculated with an average of three results [31].

### 2.5. Quantitative Detection of Biofilm Formation

The biofilm inhibitory activity of Se-NPs was assessed using the microtiter plate (MTP) technique in 96-well flat-bottom polystyrene microtiter plates and clinical isolates (*S. aureus* ATCC 29213, *P. aeruginosa* PAO1, and MDR *K. pneumonia*) according to Ruchi, et al. [32]. After 1:100 further dilution with a new medium together with control organisms with and without 0.25 and 0.5 MIC of Se-NPs, each well was filled with 200 μL of bacterial suspension in trypticase soy broth with 1 percent glucose equivalent to 0.5 McFarland. After that, the plate was incubated at 37 °C for 24 h. Then, phosphate buffer saline utilizing mild tapings (300 μL) was used for rinsing each well three times. By subjecting them to heat at 60 °C for 60 min, the remaining attaching bacteria were heat fixed. In each well, a crystal violet stain (150 μL) was applied. The surplus stain was then removed and the plate was cleaned after 15 min. For each well, 150 μL of ethanol (95%) was added and then the OD of stained adhering bacterial films was read (492 nm and 630 nm) after 30 min. The test was carried out three times and the results have been averaged. The equation [(Ac − At)/Ac] × 100 was used to calculate the percentage (%) of bacterial biofilm inhibition, where Ac was an average of six replicates of light absorption values at the wavelength (492 nm and 630 nm) of the negative controls and At was an average of six replicates of light absorption values at the wavelength (492 nm and 630 nm) of the samples [33]. Finally, an inverted microscope was used for capturing photos, showing inhibition in the bacterial biofilm.

## 3. Results and Discussion

### 3.1. Green Biosynthesis of Se-NPs Using OPW

**Figure 1:**
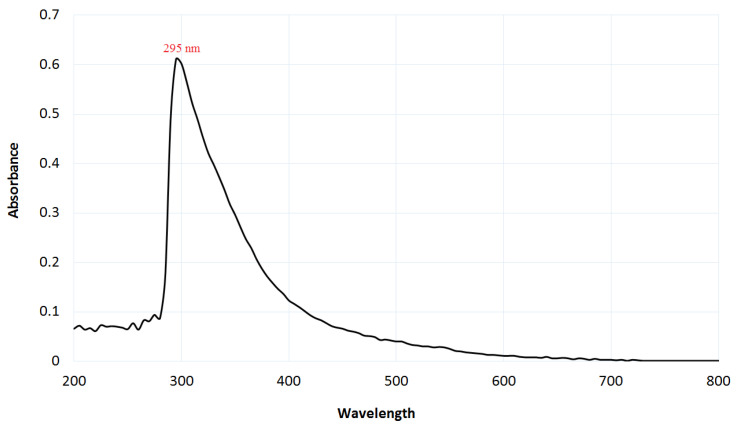
UV–vis spectrum of Se-NPs synthesized from OPW extract.

Plant materials are usually considered sustainable, environmentally friendly, and non-toxic, and highly valued due to their applications in biomedical, nutrition, and nanotechnology. Green plant-based nanoparticle production has gained popularity in recent decades as a potential alternative to chemical and physical approaches [34]. Orange peel extract (OPE) is rarely employed in the production of Se-NPs, indicating the originality of our work on Se-NPs synthesis from orange peel waste (OPW). The production of Se-NPs was shown by the formation of a reddish color in the solution because of the OPW extract interacting with selenite, showing that the ingredients present in the OPW extract could reduce these ions and change them to Se-NPs. The OPEwas employed as both a reductive and stabilizing agent to generate eco-friendly Se-NPs. By using OPW, Na_2_SeO_3_ was bio-reduced to Se-NPs, as shown by a progressive shift in the solution color from pale-yellow to deep red, indicating Se-NPs’ biosynthesis. However, the color of the control did not change. Alvi et al. [35] used citrus fruit extract to make Se-NPs. Phenolic compounds, flavonoids, limonene, and essential oils are among the bioactive substances found in orange peel (OP) [36]. The formation of Se-NPs was confirmed by a similar color shift from a yellow color to a reddish color. Figure 1 illustrates the UV analysis of OPW -synthesized Se-NPs, which reveals a significant peak at 295 nm.

Due to the formation of the Se-NPs’ surface-plasmon resonating (SPR) signal that may have been shown as a wide excitation wavelength (*λ* max) in the range of a wavelength of 270–400 nm, the creation of Se-NPs could be validated using the UV–Vis spectrophotometer [16,37,38,39]. Se-NPs have a prominent peak in their UV–Vis spectrum of about 295 nm, which is indicated to spherical Se-NPs [16,37,38]. The resulting green synthesis of Se-NPs is easy, environmentally safe, and cost-effective, and the NPs produced are non-toxic and have a good stability. Several main areas of focus aqueous extracts of various plant sections to synthesize Se-NPs have been published [40,41].

### 3.2. Characterization of Se-NPs

#### 3.2.1. Fourier-Transform Infrared Spectroscopy (FTIR)

**Figure 2:**
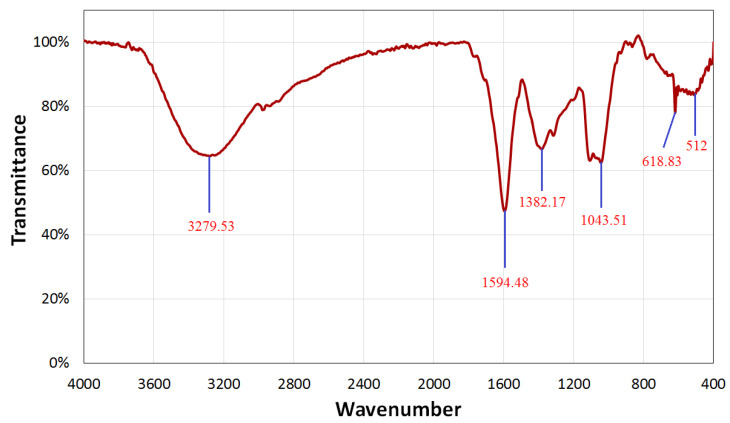
FTIR spectrum of Se-NPs synthesized from OPW extract.

FT-IR spectroscopic research was also carried out to validate the possible function of OPW extract in Se-NPs’ biosynthesis. By detecting the excitations of chemical bonds, FT-IR can determine the functional groups that are present on the surface of Se nanoparticles. The molecular data gained makes it easier to determine structural and conformational changes in the coordinating identity functional groups on Se nanoparticle surfaces. The interaction of a capping agent from the OPW extract with Se-NPs was indicated by wave numbers at 3279.53 cm^−1^, 1594.48 cm^−1^, 1382.17 cm^−1^, 1043.51 cm^−1^, 618.83 cm^−1^, and 512 cm^−1^, as shown in Figure 2.

The lines in the spectra at 3279.53 cm^−1^ correspond to O-H stretch vibrations, suggesting that OPW extract contains alcohol and phenol groups [35]. The peak in the spectra corresponds to N-C– and –C-C stretching and reveals the presence of proteins at 1594.48 cm^−1^. The spectra at 1382.17 cm^−1^ were attributed to the –N-H stretch resonance seen in the amide bonds of the proteins. The stretch vibrations of the proteins –NH and –CN corresponded to the spectra at 1382.17 cm^−1^ and 1043.51 cm^−1^, respectively. The FTIR sections of the spectra of Se-NPs showed peaks at 618.83 cm^−1^ and 512 cm^−1^, which were attributed to the binding of Se-NPs with biomaterials prepared by the OPW extract. Carbohydrates and proteins were shown to be the most abundant on the surface of Se-NPs according to FTIR analyses. The peaks’ variations suggest that the organic constituents in the extract of OPW successfully supported the formation of Se-NPs via the reduction process and may help protect Se-NPs from aggregating and hence maintain their long-term stability [14,35].

#### 3.2.2. X-ray Diffraction (XRD)

**Figure 3:**
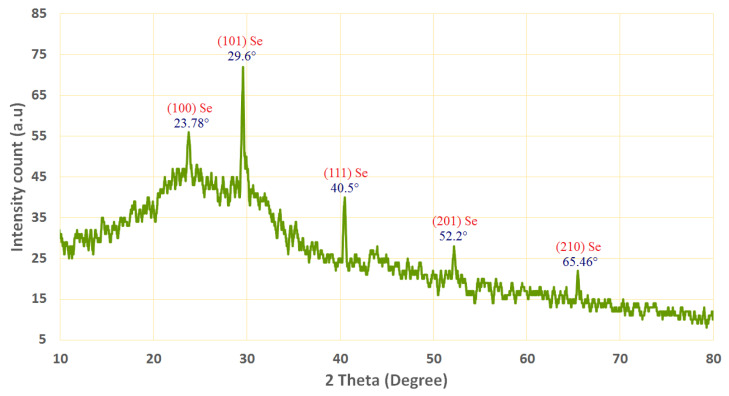
XRD pattern of Se-NPs synthesized by OPW.

XRD analysis was used to examine the crystal structure and phase of the produced Se-NPs. Figure 2 shows the XRD pattern of the produced Se-NPs. The absence of distinctive peaks for the initial precursors is plainly visible in the pattern. Figure 3 shows the XRD-diffraction peaks of Se-NPs and displays the diffraction characteristics regarding 2θ at 23.78°, 29.6°, 40.5°, 52.2°, and 65.46°, which represented the Bragg’s reflections at (100), (101), (111), (201), and (210), respectively.

All the area peaks were similar to the Joint Committee on Powder Diffraction Standards (JCPDS) of Se-NPs with the standarddcard JCPDS, file no. 06-0362 [42]. In accordance with our results, refs. [16,43,44] reported that the successful fabrication of the crystallite, monoclinic phase Se-NPs at the same XRD diffraction planes utilized metabolites of plant extracts. The XRD results indicated that the formed Se-NPs were highly crystalline for better application.

#### 3.2.3. Transmission and Scanning Electron Microscopy

**Figure 4:**
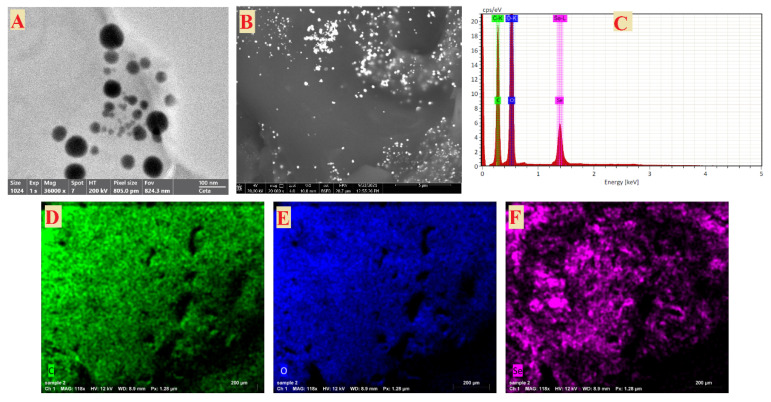
TEM image (**A**), SEM image (**B**), elemental analysis (**C**), and SEM/EDX mapping analysis (**D**–**F**) of Se-NPs synthesized by OPW.

The TEM picture indicated that the generated Se-NPs were spherical and polydisperse, and had sizes in the range of 16–95 nm, as shown in Figure 4A. As shown in Figure 4B, the SEM was used to evaluate the surface morphology and particle size of Se-NPs. The Se-NPs had a virtually spherical form. The size of the Se-NPs was made from seed extract in a liquid suspension at room temperature in the ranges of 50 to 150 nm [45]. Shakibaie et al. also generated sphericalm Se-NPs with the highest frequency of 120–140 nm inside the range of 80–220 nm [46]. The previous results showed the sizes of Se-NPs as ranging from 100 to 500 nm [47], which gives preference to the Se-NPs size that was achieved in this study. EDX analysis was used to determine the elemental composition of the Se-NPs powder [48]. In the Se-NPs, the EDX spectra revealed the existence of several well-defined elements associated with selenium [Se], oxygen [O], and carbon [C] components, as shown in Figure 4C. The Se-NPs may be surrounded by carbon and oxygen peaks [40]. The carbon [C] and oxygen [O] in the mapping of the OPW extract, different from the selenium [Se] map, indicates the creation of Se-NPs (Figure 4D–F). The peaks of carbon and oxygen may be surrounding the Se-NPs. These results are in agreement with [49,50].

### 3.3. Antibacterial Activity

Commercial antibiotics have been widely used, which has led to an increase in multidrug-resistant bacteria. Nanoparticles are a promising approach, particularly in the treatment of chronic and nosocomial infections, for antimicrobial medicines. Nanoparticles can be defined by their major benefits as antibacterial agents since they can operate many mechanisms during bacteria cannot acquire resistance to these stated action mechanisms, in contrast to commercial antibiotics [51]. Green biosynthesized Se-NPs were tested for antibacterial activity in this investigation, as indicated in Table 1.

The results revealed that Se-NPs exhibited potential antibacterial activity against both Gram-positive and negative bacteria. Moreover, the results illustrated that *S. aureus* ATCC 29213 was the most sensitive bacteria to Se-NPs at 1000 µg/mL, where the inhibition zone was 35 mm and the MIC was 25 µg/mL. In addition, MDR *E. coli*, MDR *S. aureus*, MDR *P. aeruginosa*, and MDR *K. pneumonia* were all affected by Se-NPs, with inhibition zones of 33, 33, 31, and 28 mm, respectively. *P. aeruginosa* PAO1, on the other hand, was the least susceptible bacterium to Se-NPs, with an inhibition zone of 25 mm. These results are in agreement with Huang, et al. [52] who found that MRSA is more sensitive to the antibacterial activity of the Se-NPs at concentrations more than or equivalent to 25 µg/mL. These data clearly show that MRSA is more susceptible to the antibacterial effects of Se-NP [52]. In our study, the biosynthesized Se-NPs exhibited stronger efficacy toward Gram-positive organisms. This can be ascribed to a major change in the nature of the bacterial walls, including numerous holes and thin peptidoglycan layers. Many studies have reported similar findings [52,53,54]. Additionally, orange peel has been used alone as a control sample and did not show any antibacterial activity.

Gram-negative bacteria, MDR *K. pneumoniae*, and MDR *E. coli* were the most responsive to Se-NPs, whereas *P. aeruginosa* PAO1 with a MIC of 125 µg /mL showed the least activity. Filipovi, Ujak, Milenkovi, Zheng, Liverani, Boccaccini, and Stevanovi [51] all agree with these findings, which found that *K. pneumoniae* was most susceptible to Se-NPs, whereas *P. aeruginosa* PAO1 showed the least amount of action. Additionally, Cremonini, et al. [55] recorded that Se-NPs had a MIC of 128 µg/mL against *P. aeruginosa* PAO1. Mechanisms of Se-NPs are attributed to ROS production, cell barrier interaction (cell-wall rupture and permeability change), protein and DNA synthesis inhibition, metabolic gene expression, and so on [56]. The antibacterial action is very frequently linked to ROS generation with metal-based nanoparticles (hydrogen peroxide, hydroxyl radicals, and superoxide anions). Several studies reported that Se-NPs can produce ROS [57,58]. These forms of ROS may further impede the replication of DNA and amino acid, as well as destroy the cell membrane of bacteria [59].

### 3.4. Antibiofilm Activity

**Figure 5:**
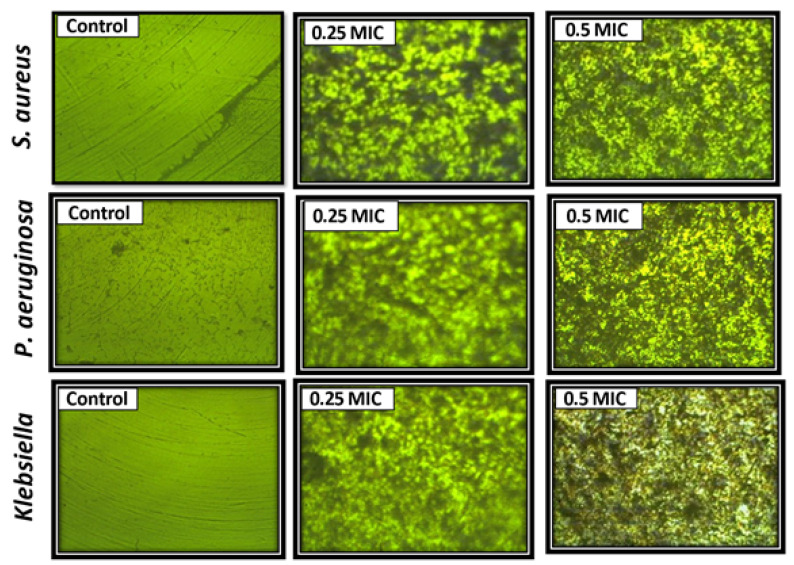
Light-inverted micrographs proved the inhibitory activity of Se-NPs (200×).

The effect of Se-NPs on the biofilm production from *S. aureus* ATCC 29213, *P. aeruginosa* PAO1, and MDR *K. pneumonia* was assessed, as shown in Table 2 and Figure 5.

The results illustrated that Se-NPs at 0.25 and 0.5 MIC decreased the biofilm significantly. The largest inhibition of the biofilm was noticed in MDR *K. pneumonia*, which was 62 and 92% at 0.25 and 0.5 MIC, respectively. Additionally, the inhibition percentage of the biofilm produced from *S. aureus* ATCC 29213 was 62.5% and 88% at 0.25 and 0.5 MIC, respectively. On the other hand, the biofilm of *P. aeruginosa* PAO1 was the least affected. The variation in cells’ numbers in culture is the first as well as the most notable observation shown after exposure to nanoparticles. Therefore, the light-inverted microscope could be used to monitor the changes in cells’ numbers dependent on the Se-NPs exposure dose. As shown in Figure 5, by raising the concentration of Se-NPs, the number of bacterial cells decreases. The biofilm structure provides several ways to resist and tolerate bacteria, thereby increasing antimicrobial activity and the immunological system of the host, and thus boosting the elimination of infections such as *S. aureus* [60]. Given the results of an antibiofilm activity, Se-NPs may be regarded as a prospective option for biofilm-formation inhibition. This inhibition has been highly documented in biofilms derived from MDR *K. pneumonia* and *S. aureus* ATCC 29213. At concentrations of 6.25–12.5 µg/mL, considerable suppression was observed, with biofilm development reduced by more than 50%. These findings are similar to Filipovi, Ujak, Milenkovi, Zheng, Liverani, Boccaccini, and Stevanovi [50], who discovered that a concentration of 6.4 µg/mL caused significant inhibition (*p* < 0.01; *p* < 0.001) in all tested strains, lowering output by more than 50%. Furthermore, Se-NPs were evaluated against different clinical isolates of *S. aureus* and showed significant inhibition at 2 µg/mL (41%) and 4 µg/mL (58%) [46]. It should be noted that the antibiofilm action of Se-NPs revealed in this work (Figure 5) was demonstrated by staining the entire biofilm biomass and extracellular polymer substances (EPS), which may be overproduced by biofilm units under stress conditions [61]. For this reason, the light-inverted microscope was used to monitor the changes in cells’ numbers dependent on the Se-NPs exposure dose. In the control experiment, the green fluorescence symbolizes living bacteria, and when the concentration of Se-NPs increased, the viability of bacteria decreased, as shown in Figure 5.

## 4. Conclusions

In the current study, orange peel waste (OPW) was used for selenium nanoparticles’ (Se-NPs) biosynthesis through a green and eco-friendly method for the first time. Characterization results revealed that biosynthesized Se-NPs were highly crystalline, spherical, and polydisperse, and had sizes in the range of 16–95 nm. Moreover, Se-NPs exhibited potential antibacterial activity against pathogenic multidrug-resistant Gram-positive and negative bacteria. Likewise, the biosynthesized Se-NPs had a promising antibiofilm activity, where the largest inhibition of biofilm was noticed in MDR *K. pneumonia*. Eventually, biosynthesized Se-NPs will have promising antibacterial and antibiofilm activity against multidrug-resistant bacteria and can be used in the medical field.

## Figures and Tables

**Table 1:** Antimicrobial activity of biosynthesized Se-NPs.

Bacterial Strain	Se-NPs		Ciprofloxacin	Gentamycin	OPW
	IZ (mm)	MIC (µg/mL)	IZ (mm)	IZ (mm)	IZ (mm)
MDR *E. coli*	33	50	12	11	ND
MDR *P. aeruginosa*	31	100	ND *	7	ND
*P. aeruginosa* PAO1	25	125	12	15	ND
MDR *K. pneumonia*	28	50	10	12	ND
*S. aureus* ATCC 29213	35	25	15	12	ND
MDR *S. aureus*	33	25	11	12	ND

* Not detected.

**Table 2:** Antibiofilm activity of Se-NPs against bacterial pathogens.

Bacterial Strain	Inhibition %	
	0.25 MIC	0.5 MIC
***S. aureus* ATCC 29213**	62.5%	88%
***P. aeruginosa* PAO1**	44%	75.5%
**MDR *K. pneumonia***	62%	95%

## Data Availability

Not applicable.
